# Glycomimetics: Design, Synthesis, and Therapeutic Applications

**DOI:** 10.3390/molecules23071658

**Published:** 2018-07-07

**Authors:** Philippe Compain

**Affiliations:** Laboratoire d’Innovation Moléculaire et Application (LIMA), Université de Strasbourg, Université de Haute-Alsace, CNRS (UMR 7042), Equipe Synthèse Organique et Molécules Bioactives (SYBIO), Ecole Européenne de Chimie, Polymères et Matériaux, 25 rue Becquerel, 67000 Strasbourg, France; philippe.compain@unistra.fr; Tel.: +33-3-6885-2792

Glycomimetics as structurally altered analogues of sugars offer the opportunity to emulate carbohydrate activities, while circumventing their drawbacks as credible drugs [1,2,3,4,5]. Structural modifications are performed not only to enhance target affinity and selectivity, but also to improve drug-like properties, including oral bioavailability or in vivo stability. Beyond their biological interest, glycomimetics are an amazing playground for chemists. Designing new chemical structures that compete with one of the most important classes of biomolecules is indeed a strong driving force for the practitioners of the art and science of organic chemistry. To paraphrase Marcellin Berthelot, chemists, and especially organic chemists, are eager to create their own objects of study. Beyond rational thinking, a kind of quest for molecular beauty that can be achieved by means of simplicity or symmetry is part of the design process [6]. This aesthetic quest by no means excludes the search for therapeutic applications; for pharmaceutical companies, one of the main drawbacks of carbohydrates as drug candidates is indeed their structural complexity. With regards to synthetic methodologies, the relative structural complexity of glycomimetics—with a high density of functional groups and asymmetric centers—also provides a formidable testing ground for known reactions while permitting accidental discoveries in synthesis [7]. The objective of this special issue is to provide contributions that highlight the main current aspects in the field of glycomimetics, from innovative synthesis to potential therapeutic applications. Carbohydrate mimetics could indeed be designed for structural originality, tackling fundamental questions in glycobiology, or drug discovery. This special issue provides a compilation of original research articles and reviews that is representative of the current research in the field. 

Glycosylamines have been shown to be useful intermediates in the synthesis of iminosugars, which are undoubtedly one of the most attractive uses of glycomimetics reported so far [8,9]. In addition to providing the asymmetric centers and functionalities needed, *N*-glycosyl compounds are capable to react via their open-chain imine form, with a large variety of carbon nucleophiles. The aminoalditols thus obtained contain all the key elements of the structure of the final targets, including the iminosugar endocyclic nitrogen atom. After further functionalization and cyclization, iminosugar *C*-glycosides [10] are obtained. Nicolas and Martin present an overview of the most significant glycosylamine-based methodologies developed to date for the synthesis of stable iminosugars, and discuss the advantages and possible limitations associated with this synthetic strategy [11]. 

Due to the tremendous biological importance of nucleosides and glycoconjugates, many efforts have been directed towards the development of efficient strategies towards the synthesis of analogues, with preserved or new properties. Aminooxyl functionalized compounds have shown their interest as building blocks for the easy coupling of carbohydrate units via the formation of *N*-oxyamide or oxime linkages. Such synthetic approaches take advantage of the high reactivity of the oxyamine functional group. Chen and Xie provide an overview of their synthetic efforts in the field, and convincingly demonstrate the interest in aminooxyl functionalized sugars in glycoscience [12]. 

The work presented by Céspedes Dávila et al. is a further illustration of the fact that carbohydrates hold a high potential for serendipitous discoveries in organic synthesis [13]. In connection with their objective to develop an efficient thiol-click strategy for the synthesis of *neo*-oligosaccharides [14], the authors needed to prepare α-glycosyl thiols following a robust protocol, based on a TMSOTF-mediated ring opening of 1,6-anhydro sugars with (TMS)_2_S [15]. In their first attempt, the accidental presence of traces of acetone in the reaction mixture led to the formation of a dithioacetal-α,α-diglycoside product, and eventually to the development of one-pot, highly stereo-selective access to a class of disaccharide mimetics with almost no precedent. 

In the contribution of Redjdal et al. [16], α-Glycosyl thiols were also used as key building blocks for the development of an innovative synthetic methodology. Based on their expertise in organometallic chemistry, the authors synthesized a series of *N*,*S*-bis-glycosyl quinolin-2-ones by way of Buchwald—Hartwig—Migita cross-coupling. Heteroaryl-glycosides constitute a very important class of glycoconjugates found in natural products and therapeutically relevant compounds. An illustration of the biological interest of such structures is provided by Somsák et al. who reported a systematic study towards the development of glycogen phosphorylase inhibitors based on β-*C*- and *N*-glucopyranosyl derivatives of a wide range of azole type heterocycles [17]. Glycogen phosphorylase plays a key role in glycogenolysis, and as such, is a target for the development of drugs against type II diabetes.

Iminosugars are historically known as potent glycosidase inhibitors [8,9]. In their attempts to discover non-toxic insecticides or fungicides, Cardona et al. present the synthesis of new inhibitors of trehalases. These glycosyl hydrolases catalyze the breaking of the glycosidic bond in trehalose, a non-mammalian disaccharide consisting of two D-glucose molecules linked by an α,α-1,1-linkage. In the pseudodisaccharides designed as trehalose mimetics, one glucose unit was replaced by a polyhydroxylated pyrrolizidine or pyrrolidine moiety [18]. The distance between the glucosyl and the iminosugar unit was found to have a strong impact in the inhibition potency. Mena-Barragán et al. also took advantage of iminosugars to develop inhibitors of human glucocerebrosidase that act as picomolar chaperones for Gaucher disease, the most prevalent lysosomal storage disorders [19]. This group of rare genetic diseases is characterized by a deficiency of glycosidases involved in the catabolism of glycosphingolipids in the lysosome. The pharmacological chaperone therapy is based on reversible inhibitors that are able to impact the three-dimensional architecture of the misfolded but catalytically active glycosidases involved in lysosomal storage disorders. This interaction prevents their degradation by the endoplasmic reticulum-associated degradation (ERAD) pathway before trafficking to lysosomes [20]. In this counterintuitive approach, a massive enzyme is thus “rescued” by its low-molecular-weight inhibitor at subinhibitory concentrations. Mena-Barragán et al. show in their contribution that the inhibitor/chaperone balance could be controlled by predefining the reducing or non-reducing character of sp^2^-iminosugars [19]. In these compounds, the typical sp^3^ endocyclic nitrogen atom of iminosugars is replaced by a pseudoamide-type nitrogen atom. This offers the possibility of accessing stable derivatives substituted by an OH group at the pseudoanomeric position to mimic sugar-reducing ends. Carbasugars are also an important class of pharmacological chaperones. Stütz et al. report the synthesis of amine-containing cyclopentanols as hexosamine mimetics featuring the D-*galacto* configuration [21]. These compounds were found to be potent inhibitors of GH20 *N*-Acetyl D-hexosaminidases, and as such are potential pharmacological chaperones for the treatment of Tay Sachs disease.

As highlighted by all these articles, glycomimetics are an endless source for discoveries in chemistry, biology, and medicinal sciences. For chemists, they provide exciting synthetic challenges, as well as the pleasure of designing unprecedented structures that compete with a major class of biomolecules, with imagination being the only limit. For biologists, glycomimetics offer molecular probes to decipher complex biological processes, from carbohydrate-mediated recognition events to protein trafficking. For medicinal chemists, glycomimetics appear as one of the means for translating the biological activities of carbohydrates into therapeutic applications. Finally, the guest editor is grateful to all the authors, from no less than three continents, for having illustrated so well the importance of glycomimetics in their contributions to this special issue.

## References

[1] Sears P., Wong C.-H. (1999). Carbohydrate mimetics: A new strategy for tackling the problem of carbohydrate-mediated biological recognition. Angew. Chem. Int. Ed..

[2] Compain P., Martin O.R. (2001). Carbohydrate mimetics-based glycosyltransferase inhibitors. Bioorg. Med. Chem..

[3] Ernst B., Magnani J.L. (2009). From carbohydrate leads to glycomimetics drugs. Nat. Rev. Drug Discov..

[4] Chapleur Y. (1998). Carbohydrate Mimics, Concepts and Methods.

[5] Koester D.C., Dennis C., Holkenbrink A., Werz D.B. (2010). Recent advances in the synthesis of carbohydrate mimetics. Synthesis.

[6] Hoffmann R. (1990). Molecular Beauty. J. Aesth. Art Crit..

[7] Compain P. (2014). Searching for glycomimetics that target protein misfolding in rare diseases: Successes, failures and unexpected progress made in organic synthesis. Synlett.

[8] Compain P., Martin O.R. (2007). Iminosugars: From Synthesis to Therapeutic Applications.

[9] Stütz A.E. (1999). Iminosugars as Glycosidase Inhibitors.

[10] Compain P., Chagnault V., Martin O.R. (2009). Tactics and strategies for the synthesis of iminosugar C-glycosides: A review. Tetrahedron Asymmetry.

[11] Nicolas C., Martin O.R. (2018). Glycoside mimics from glycosylamines: Recent progress. Molecules.

[12] Chen N., Xie J. (2018). *O*-Amino sugars and nucleosides. Molecules.

[13] Céspedes Dávila M.F., Schneider J.P., Godard A., Hazelard D., Compain P. (2018). One-pot, highly stereoselective synthesis of dithioacetal-α,α-diglycosides. Molecules.

[14] Lepage M.L., Schneider J.P., Bodlenner A., Compain P. (2015). Towards a molecular lego approach for the diversity-oriented synthesis of cyclodextrin analogues designed as scaffolds for multivalent systems. J. Org. Chem..

[15] Zhu X., Dere R.T., Jiang J., Zhang L., Wang X. (2011). Synthesis of α-glycosyl thiols by stereospecific ring-opening of 1,6-anhydrosugars. J. Org. Chem..

[16] Redjdal W., Ibrahim N., Benmerad B., Alami M., Messaoudi S. (2018). Convergent Synthesis of *N*,*S*-bis glycosylquinolin-2-ones via a Pd-G3-XantPhos precatalyst catalysis. Molecules.

[17] Kun S., Bokor E., Sipos A., Docsa T., Somsák L. (2018). Synthesis of new *C*- and *N*-β-d-glucopyranosyl derivatives of imidazole, 1,2,3-triazole and tetrazole, and their evaluation as inhibitors of glycogen phosphorylase. Molecules.

[18] D’Adamio G., Forcella M., Fusi P., Parenti P., Matassini C., Ferhati X., Vanni C., Cardona F. (2018). Probing the influence of linker length and flexibility in the design and synthesis of new trehalase inhibitors. Molecules.

[19] Mena-Barragán T., Isabel García-Moreno M., Sevšek A., Okazaki T., Nanba E., Higaki K., Martin N.I., Pieters R.J., García Fernández J.M., Ortiz Mellet C. (2018). Probing the inhibitor versus chaperone properties of sp^2^-iminosugars towards human β-glucocerebrosidase: A picomolar chaperone for Gaucher disease. Molecules.

[20] Parenti G. (2009). Treating lysosomal storage diseases with pharmacological chaperones: From concept to clinic. EMBO Mol. Med..

[21] Weber P., Nasseri S.A., Pabst B.M., Torvisco A., Müller P., Paschke E., Tschenutter M., Windischhofer W., Withers S.G., Stütz A.E. (2018). Potent GH20 *N*-Acetyl-β-d-hexosaminidase inhibitors: *N*-Substituted 3-acetamido-4-amino-5-hydroxymethyl-cyclopentanediols. Molecules.

